# [Retracted] MicroRNA 217 inhibits cell proliferation and enhances chemosensitivity to doxorubicin in acute myeloid leukemia by targeting KRAS

**DOI:** 10.3892/ol.2026.15482

**Published:** 2026-02-09

**Authors:** Yi Xiao, Taoran Deng, Changliang Su, Zhen Shang

Oncol Lett 13: 4986–4994, 2017; DOI: 10.3892/ol.2017.6076

Following the publication of this paper, it was drawn to the Editor's attention by a concerned reader that, regarding the flow cytometric data shown in Figs. 5 and 8D, all four sets of quadrant plots were strikingly similar, suggesting that the same data had been included in these figures to show the results of differently performed experiments. Moreover, upon performing an independent analysis of the data in this paper in the Editorial Office, it came to light that the majority of these data reappeared in different guises in three subsequent publications that were published in different journals by different authors at different research institutes.

Given the duplication of the abovementioned data within this paper, and its subsequent reappearance in various other journals, the Editor of *Oncology Letters* has decided that this paper should be retracted from the Journal on account of a lack of confidence in the scientific integrity of the study. The authors were asked for an explanation to account for these concerns, but the Editorial Office did not receive a reply. The Editor apologizes to the readership for any inconvenience caused.

